# Transcriptomic analysis reveals that methyl jasmonate confers salt tolerance in alfalfa by regulating antioxidant activity and ion homeostasis

**DOI:** 10.3389/fpls.2023.1258498

**Published:** 2023-09-14

**Authors:** YanLing Yin, TianHui Yang, Shuang Li, Xiaoning Li, Wei Wang, ShuGao Fan

**Affiliations:** ^1^ School of Resources and Environmental Engineering, Ludong University, Yantai, Shandong, China; ^2^ Institute of Animal Science, Ningxia Academy of Agriculture and Forestry Sciences, Yinchuan, China

**Keywords:** MeJA, salt tolerance, antioxidant capacity, ion homeostasis, *Medicago sativa*

## Abstract

**Introduction:**

Alfalfa, a globally cultivated forage crop, faces significant challenges due to its vulnerability to salt stress. Jasmonates (JAs) play a pivotal role in modulating both plant growth and response to stressors.

**Methods:**

In this study, alfalfa plants were subjected to 150 mM NaCl with or without methyl jasmonate (MeJA). The physiological parameters were detected and a transcriptomic analysis was performed to elucidate the mechanisms underlying MeJA-mediated salt tolerance in alfalfa.

**Results:**

Results showed that exogenous MeJA regulated alfalfa seed germination and primary root growth in a dose-dependent manner, with 5µM MeJA exerting the most efficient in enhancing salt tolerance. MeJA at this concentration elavated the salt tolerance of young alfalfa seedlings by refining plant growth, enhancing antioxidant capacity and ameliorating Na+ overaccumulation. Subsequent transcriptomic analysis identified genes differentially regulated by MeJA+NaCl treatment and NaCl alone. PageMan analysis revealed several significantly enriched categories altered by MeJA+NaCl treatment, compared with NaCl treatment alone, including genes involved in secondary metabolism, glutathione-based redox regulation, cell cycle, transcription factors (TFs), and other signal transductions (such as calcium and ROS). Further weighted gene co-expression network analysis (WGCNA) uncovered that turquoise and yellow gene modules were tightly linked to antioxidant enzymes activity and ion content, respectively. Pyruvate decar-boxylase (PDC) and RNA demethylase (ALKBH10B) were identified as the most central hub genes in these two modules. Also, some TFs-hub genes were identified by WGCNA in these two modules highly positive-related to antioxidant enzymes activity and ion content.

**Discussion:**

MeJA triggered a large-scale transcriptomic remodeling, which might be mediated by transcriptional regulation through TFs or post-transcriptional regulation through demethylation. Our findings contributed new perspectives for understanding the underneath mechanisms by which JA-mediated salt tolerance in alfalfa.

## Introduction

Soil salinization is progressively becoming a detrimental constraint on plant growth and productivity (Singh, 2021). It is estimated that over 20% of irrigated land is currently influenced by salinity, and this is expected to persistently expand due to warming temperatures, irrigation practices, and soil degradation (Sahab et al., 2021). Among the different types of soil salts, NaCl is the most soluble and common. High levels of Na^+^ in the rhizosphere can cause detrimental effects on plants, leading to photosynthetic inhibition, enzyme deactivation, and metabolic alterations (Yang and Guo, 2018). These changes are the results of disruption in plant–water balance (osmotic stress), redox, and ionic homeostasis (Van Zelm et al., 2020). Moreover, to cope with stressful conditions, plants undergo growth and developmental arrest and prioritize defense responses, resulting in physiological and developmental alterations (Lozano-Durán and Zipfel, 2015).

Plants are sessile, rendering them incapable of escaping the environmental salinity. Therefore, they must adopt strategies to confront the adverse consequences of salt stress by modifying their physical attributes and developmental patterns (Heydarian et al., 2018; Nefissi Ouertani et al., 2021). Plant response to salt stress is a temporally and spatially separated two-phase pattern, comprising earlier osmotic stress response and the late ion toxicity response (Munns and Tester, 2008; Yang et al., 2019; Van Zelm et al., 2020). When plants are subjected to salt stress, Ca^2+^ is stimulated as the initial signal molecule (Yang et al., 2019). Elevation in intracellular Ca^2+^ level triggers the expression of genes encoding Ca^2+^ sensors including calmodulin (CaM), CaM-like protein (CML), calcineurin B-like protein (CBL), and calcium-dependent protein kinase (CDPK) (Wang et al., 2018; Yue et al., 2022). Subsequently, genes related to osmoregulation, antioxidants, and ion exclusion/sequestration responded to salt stimuli. Decreased osmotic pressure under salt stress reduces water availability, ultimately inducing osmotic stress (Van Zelm et al., 2020). Plants synthesize osmolytes like proline, soluble sugars, and organic acids, which serve to uphold cellular volume and turgor (Flowers and Yeo, 1995). Genes related to proline biosynthesis, such as *P5CS*, have been documented to be triggered by salt stress. Overexpressed *PvP5CS* in switchgrass led to an enhancement in plant salt tolerance (Silva-Ortega et al., 2008; Guan et al., 2020). Salt stress triggers the expression of genes encoding ion transporters such as salt overly sensitive (SOS), Na^+^/H^+^ exchanger (NHX), and high-affinity Na^+^/K^+^-permeable transporter (HKT) (Hauser and Horie, 2010). These transporters are responsible for transferring Na^+^ and K^+^, thus maintaining ionic balance, as well as reducing the Na^+^ accumulation (Saddhe et al., 2021). Reactive oxygen species (ROS) burst happens when the cells suffer from osmotic stress or ion toxicity, causing oxidative damage. ROS, including hydrogen peroxide (H_2_O_2_), superoxide anion (O^2−^), and hydroxy radical (OH^−^) accumulated in apoplast and organelle rapidly under salt stress, disturbing the balance of cell redox homeostasis (Miller et al., 2010). Subsequently, the antioxidant systems are activated to reduce the production or induce the scavenging of ROS (He et al., 2017). ROS scavenging enzymes, such as superoxide dismutase (SOD), peroxidase (POD), and catalase (CAT), are triggered under salt stress (Singh et al., 2022). Moreover, non-enzymatic antioxidants such as ascorbic acid (ASA), glutathione (GSH), and carotene accumulate under salt stress to quench the ROS (Ahmad et al., 2019). Moreover, MAPK cascades and some second messengers are engaged in mitigating the adverse effects of salt stress (Wei et al., 2022). Many salt stress-responsive transcription factors (TFs; e.g., WRKYs, NACs, and MYBs) are induced by stress to govern the transcription of specific target genes through their interaction with promoter regions (Shah et al., 2021).

Changes in phytohormone levels are downstream signals that significantly contribute to salt stress resistance (Peleg and Blumwald, 2011). The regulation in biosynthesis, metabolism, and signal transduction of phytohormones also leads to the alteration of gene expression levels related to plant salt-adaptive response (Yu et al., 2020). Jasmonates (JAs) including jasmonic acid (JA) and its offshoots such as methyl jasmonate (MeJA) are derivatives of oxidized fatty acids and act as ubiquitous regulators in plant growth and defense (Ali and Baek, 2020). Previous studies have intensively demonstrated the roles of JAs in plant immune responses to pathogens and insects (Du et al., 2017). Moreover, JAs also have attracted attention as protectors against abiotic stress (Ali and Baek, 2020). However, the functions of JAs in salt stress responses remain unclear and even controversial. Many transcriptomic analyses have indeed corroborated the upregulation of genes associated with JA biosynthesis in salt-stressed roots (Jiang and Deyholos, 2006; Geng et al., 2013). Physiology and biochemistry studies documented that the endogenous JA was accumulated under salt stress in tomato (Pedranzani et al., 2013), sweet potato (Zhang et al., 2017), and wheat (Zhu et al., 2022). Exogenous application of JAs significantly mitigated the salt-stressed symptoms in *Triticum aestivum* (Qiu et al., 2014), *Vitis vinifera* (Karimi et al., 2022), and *Nitraria tangutorum* (Gao et al., 2021). Deficiency in A biosynthesis decreased the salt tolerance of maize shoots (Ahmad et al., 2019) and tomato (Abouelsaad and Renault, 2018). Furthermore, overexpression of JA biosynthesis genes of rice in tobacco effectually promoted its salt tolerance (Asfaw et al., 2022). These findings confirmed the beneficial regulatory functions of JA in enhancing plant resilience to salt stress. Furthermore, it has been revealed that JA-improved salt tolerance was involved in the enhancement of antioxidant ability (Gao et al., 2021), rebuilding of ion homeostasis (Ahmad et al., 2019), and striking a balance between plant growth and resistance (Gao et al., 2021). However, mutation of the JA biosynthetic enzyme, ALLENE OXIDE CYCLASE (AOC) in rice, which showed reduced endogenous JA content, enhanced salt tolerance (Hazman et al., 2015). Similarly, overexpression of the master TF of JA signal, MYC2, weakened the salt resilience of *Arabidopsis* by repressing the transcription of *CAT2* (Song et al., 2021). These investigations suggest that JA participates in the salt stress responses in a positive or negative manner for different species. The underlying mechanisms of JA-mediated salt tolerance need further research.

Alfalfa is a worldwide cultivated forage and is famous for its high value in forage quality and ecological protection. Like most crop plants, alfalfa is a glycophyte with neutral salt tolerance (Singer et al., 2018). Alfalfa plants challenged by severe salt stress show decreases in both root and shoot growth, as well as nutritional value (Stritzler et al., 2018). Exploring the regulatory framework linked to salt stress in alfalfa holds significant importance. Here, we hypothesize that the salt stress responses of alfalfa are mediated by JAs. In this study, we detected the growth and physiological parameters of NaCl-stressed alfalfa with or without MeJA treatment and performed a genome-wide transcriptomic analysis to uncover the differentially expressed genes (DEGs) altered by MeJA and NaCl. Subsequently, we carried out PageMan analysis and weighted gene co-expression network analysis (WGCNA) to reveal the signaling pathways and hub genes regulated by MeJA. The results of this study will illuminate the molecular mechanisms by which JA mediated salt tolerance in alfalfa and provide the potential to offer novel avenues for enhancing the salt tolerance of this crop.

## Materials and methods

### Plant material and experimental design

Cultivated alfalfa “XinJiangDaYe” used in this research was obtained from the Ningxia Academy of Agriculture and Forestry Sciences. For the germination assay, 100 seeds were germinated in Petri dishes containing 0, 5, 10, 50, and 100 μM of MeJA with or without 150 mM of NaCl. The germination rate and root length were measured on the seventh day of post-treatment. For the young seedling treatments, 3-week-old young seedlings were transplanted into a half-strengthened Hoagland nutrient solution (Hoagland and Arnon, 1950). After adapting growth for 1 week, plants were exposed to 1/2 Hoagland nutrient solution with 5 μM of MeJA and 150 mM of NaCl separately or combined. Physiological parameter detections and transcriptomic analysis were performed after treatment for 24 h. Growth-related characteristics were determined after treatment for 1 week.

### Measurement of plant height, root length, and plant biomass

Plant height was measured as the vertical distance from the cotyledonary node to the apical bud using a calibrated ruler. Root length was determined as the length from the tips of the longest root to the cotyledonary node. Fresh weight was measured using a non-destructive method. The entire plants were carefully harvested and separated for the shoot and root parts, with the cotyledon node serving as the demarcation point between these sections. Shoots and roots were washed three to five times using deionized water and dried with absorbent paper. The fresh weight of shoots and roots was then weighed using an electronic balance.

### Detection of antioxidant enzyme activity

Crude enzymes were extracted from 0.1-g root samples in ice-cold phosphate buffer saline (PBS; pH 7.8, 50 mM) containing 0.2 mM of EDTA, 2 mM of l-ascorbic acid, and 2% (w/v) polyvinylpolypyrrolidone. The supernatant obtained through centrifugation was used to assess enzyme activity following the method outlined by Xia et al. (2009). SOD activity was measured by the reduction of nitroblue tetrazolium (NBT) by superoxide anions. CAT activity was determined by quantifying the rate of H_2_O_2_ decomposition. The enzyme activities were quantified by dividing the enzyme activity obtained by the corresponding protein concentration. The protein content in the supernatants was determined using a Bradford Protein Assay Kit (Sigma-Aldrich, Darmstadt, Germany).

### Measurement of H_2_O_2_ and proline contents

H_2_O_2_ content was measured according to Willekens et al. (1997) with slight modifications. Briefly, a 0.1-g alfalfa root sample was homogenized utilizing 1 M of HClO_4_. Subsequent to centrifugation at 6,000 *g* for 10 minutes, adjustment of the supernatant pH to the range of 6.0–7.0 was executed through the introduction of 4 M of KOH. Mixing equal volumes of the resulting supernatant with a reaction buffer containing 100 mM of potassium acetate (pH 4.4) and 1 mM of 2,2′-hydra-bis(3-ethylbenzothiazolin-6-sulfonic acid) ensued. The absorption at 412 nm was observed for both instances with the addition of POD enzyme and without it. The disparity in absorbance between these two conditions represented the H_2_O_2_ content. Precise quantification of H_2_O_2_ content was achieved by referencing a standard curve based on a gradient of H_2_O_2_ concentrations.

Proline content was detected based on a previously reported protocol with minor modification (Bates et al., 1973). Fresh root samples (0.1 g) were homogenized in 10 mL of 3% aqueous sulfosalicylic acid, and the homogenate was centrifuged at 10,000 *g* for 10 minutes. The supernatant (2 mL) was mixed with 2 mL of acid ninhydrin and 2 mL of glacial acetic acid, and the mixture was heated in a boiling water bath for 1 h. After cooling, the chromophore was extracted with 4 mL of toluene, and the absorbance of the organic layer was measured at 520 nm using a spectrophotometer. Proline concentration was calculated based on a standard curve generated using known concentrations of proline.

### Quantification of Na^+^ and K^+^

Root and leaf samples were harvested and subjected to a three-times washing using deionized water. The samples were first heated to 105°C for 30 minutes to deactivate the enzymes, followed by a drying process at 75°C for 72 h. Dried samples at 0.1 g were prepared by digestion of plant tissues in a 5:1 mixture of nitric acid and perchloric acid (v/v). The crude extraction was filtered and then diluted to reach a final volume of 50 mL. The Na^+^ and K^+^ contents were measured using an atomic absorbance spectrophotometer equipped with a flame atomizer (Shimadzu, Kyoto, Japan; A6300).

### Detection of chlorophyll *a* fluorescence transient curve

Rapid measurement of leaf chlorophyll fluorescence transient curves was performed using a plus-amplitude modulated fluorometer (PAM2500, Heinz Walz GmbH, Pfullingen, Germany). After 1-week treatment, the third fully expanded leaves harvested from each treatment were subjected to a 30-minute dark adaptation. Then, a saturating pulse of light (3,500 mmol photons m^−2^ s^−1^) was applied to the leaves to induce maximum fluorescence (Fm). The resulting chlorophyll fluorescence emission was measured and converted into digital format, with a time resolution ranging from 10 µs to 320 ms.

### RNA isolation, cDNA library construction, and Illumina sequencing

Root samples of NaCl, MeJA (J), MeJA combined NaCl (MeJA+NaCl) treatments, and control (CK) were collected after treatment for 24 h. The total RNA was extracted using a Plant total RNA purification kit (GMbiolab Co., Ltd., Taiwan). The RNA purity and quantity were confirmed by Qubit 2.0 fluorometer, and the integrity was detected by 2100 Bioanalyzer (Agilent, Santa Clara, CA, USA).

An input material of 1 µg of RNA per sample was utilized for the preparation of RNA samples. Total mRNA was spliced randomly into short fragments of approximately 300 bp in the NEB Fragmentation Buffer. Random hexamer primers and M-MuLV Reverse Transcriptase (RNase H-) were employed to synthesize the first-strand cDNA using the short fragments as templates. Subsequently, second-strand cDNA was synthesized using DNA Polymerase I and RNase H. NEBNext adaptors with hairpin loop structure were ligated to the double-stranded cDNA fragments. Afterward, the library fragments were purified with the AMPure XP system (Beckman Coulter, Beverly, MA, USA) to obtain 250–300-bp cDNA fragments. The fragments were amplified by PCR and purified through AMPure XP beads to create the final library. The quality of the library was assessed, and the prepared cDNA library was then subjected to high-throughput sequencing on the Illumina sequencing platform by Metware Biotechnology Co., Ltd. (Wuhan, China).

### Mapping reads to the reference genome and conducting analysis of expression levels

Quality filtering of raw reads was performed using FASTp. Adapter-containing reads and low-quality reads (Phred quality value < 20, containing poly-N) were removed to generate the clean reads. The subsequent analysis was performed based on clean reads. Then, the clean reads were mapped to the alfalfa XinJiangDaYe genome using HISAT version 2.1.0 with default parameters. The transcription level was estimated by FPKM (Fragments Per Kilobase of transcript per Million fragments mapped).

### DEG identification and PageMan analysis

DEGs in the transcriptome data were identified through the utilization of the DESeq2 package (v1.22.1) within the R programming environment. Raw read counts were used as input data for the analysis. DEGs were identified using criteria where the absolute value of log_2_ (fold change (FC)) ≥ 1, and the false discovery rate (FDR) < 0.05.

Enrichment analysis of the DEGs was performed using the PageMan software (v3.0.0), which is a commonly used tool for functional analysis of large-scale omics data in plants. The log_2_ (FC) of NaCl vs. CK and that of NaCl+MeJA vs. CK were input into PageMan, and the significantly enriched BINs were identified using the Wilcoxon statistical test with default parameters provided by PageMan.

### Weighted gene co-correlation network analysis

The construction of co-expression networks and the identification of modules comprising closely correlated genes were carried out using the WGCNA R package. A signed network was constructed using the normalized FPKM of DEGs. The dynamic Tree Cut algorithm was employed to establish modules, ensuring a minimum size of 30 genes for each module. Module eigengenes were calculated, and module–trait associations were tested using Pearson’s correlation analysis with physiological trait data. The modules that exhibited the highest correlation coefficient with specific physiological traits at a significance level of *p* < 0.05 were selected to construct the co-expression network. Hub genes within the co-expression network were identified through topological overlap measures and were prioritized using module membership and gene significance.

### qRT-PCR analysis

qRT-PCR was employed to validate the RNA-Seq data. Total RNA of 500 ng was reverse-transcribed into cDNA using a first-strand cDNA synthesis kit (Toyobo, Tokyo, Japan; FSQ-201). The expression levels of the eight hub genes were quantified by qRT-PCR using a real-time PCR system (StepOnePlus, Applied Biosystems, Foster City, CA, USA) and a SYBR green master mix (Takara, Mountain View, CA, USA). The relative expression levels of target genes were determined by the 2^−ΔΔCt^ method. The housekeeping gene *MsACTIN* was used as the internal control. Primers for specific genes are presented in **Table S1**. The experiment comprised three biological replicates for each treatment.

### Statistical analysis

Statistical analysis was conducted using SPSS 20 software. A one-way ANOVA was performed to identify any significant differences among groups at a significance level of *p* < 0.05. *Post-hoc* Tukey’s test was used to determine the significant differences among groups.

## Results

### Effects of MeJA on plant growth of NaCl-stressed alfalfa

**Figure 1:**
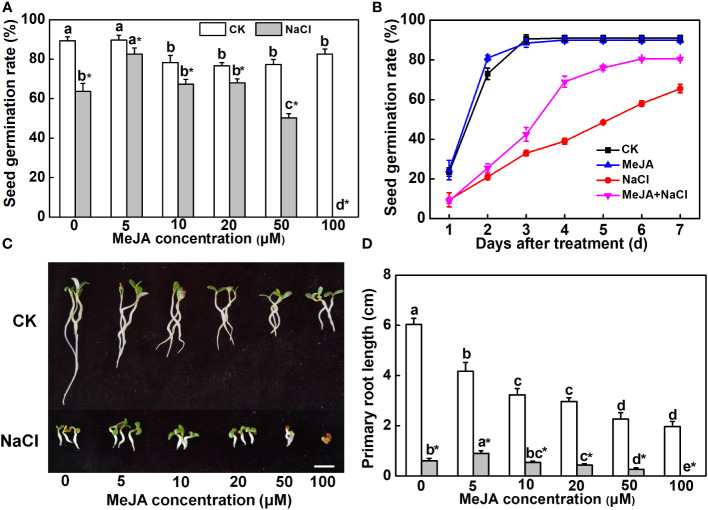
Effects of methyl jasmonate (MeJA) application on seed germination and primary root growth. **(A)** Seed germination rate after 7-day treatment. Columns represent average of three biological replicates with standard deviation, and each replicate has 100 seeds. **(B)** Time-course response of seed germination. The concentration of MeJA was 5 μM. **(C)** Primary root phenotypes. Bar = 1 cm. **(D)** Primary root length. Columns with different lowercase letters indicate significant differences among treatments at *p* < 0.05 using Duncan’s test. Data represent average of three biological replicates with standard deviation, and each replicate has 10 plants. CK indicates control.

To investigate the effect of MeJA on seed germination and primary root growth under salt stress, alfalfa seeds were subjected to different concentrations of MeJA. A higher concentration of MeJA (>10 μM) slightly suppressed seed germination under normal conditions (**Figure 1A**). NaCl at a concentration of 150 mM depressed the seed germination rate by 30%, whereas 5 μM of MeJA mitigated the adverse impact of NaCl on seed germination (**Figure 1A**). However, 50 μM of MeJA exacerbated the inhibitory impact of salt stress on seed germination, and 100 μM of MeJA completely abolished the germination of salt-stressed seeds (**Figure 1A**). The exploration of time-course effects on seed germination under various treatments unveiled a significant acceleration in salt-stressed seeds subjected to 5 μM of MeJA. Impressively, these seeds achieved a 69% germination rate within a mere 4 days, surpassing the germination rate of 65.5% observed for the NaCl treatment over the entire 7-day duration (**Figure 1B**). Consequently, seeds subjected to the MeJA+NaCl treatment exhibited enhanced primary root development and cotyledon expansion compared to those treated only with NaCl, even though MeJA inhibited root elongation under non-saline conditions (**Figures 1C, D**). MeJA higher than 20 μM aggravated the negative effect of NaCl on primary root development (**Figures 1C, D**). Therefore, MeJA at 5 μM was determined as the optimal concentration for improving salt tolerance of alfalfa.

**Figure 2:**
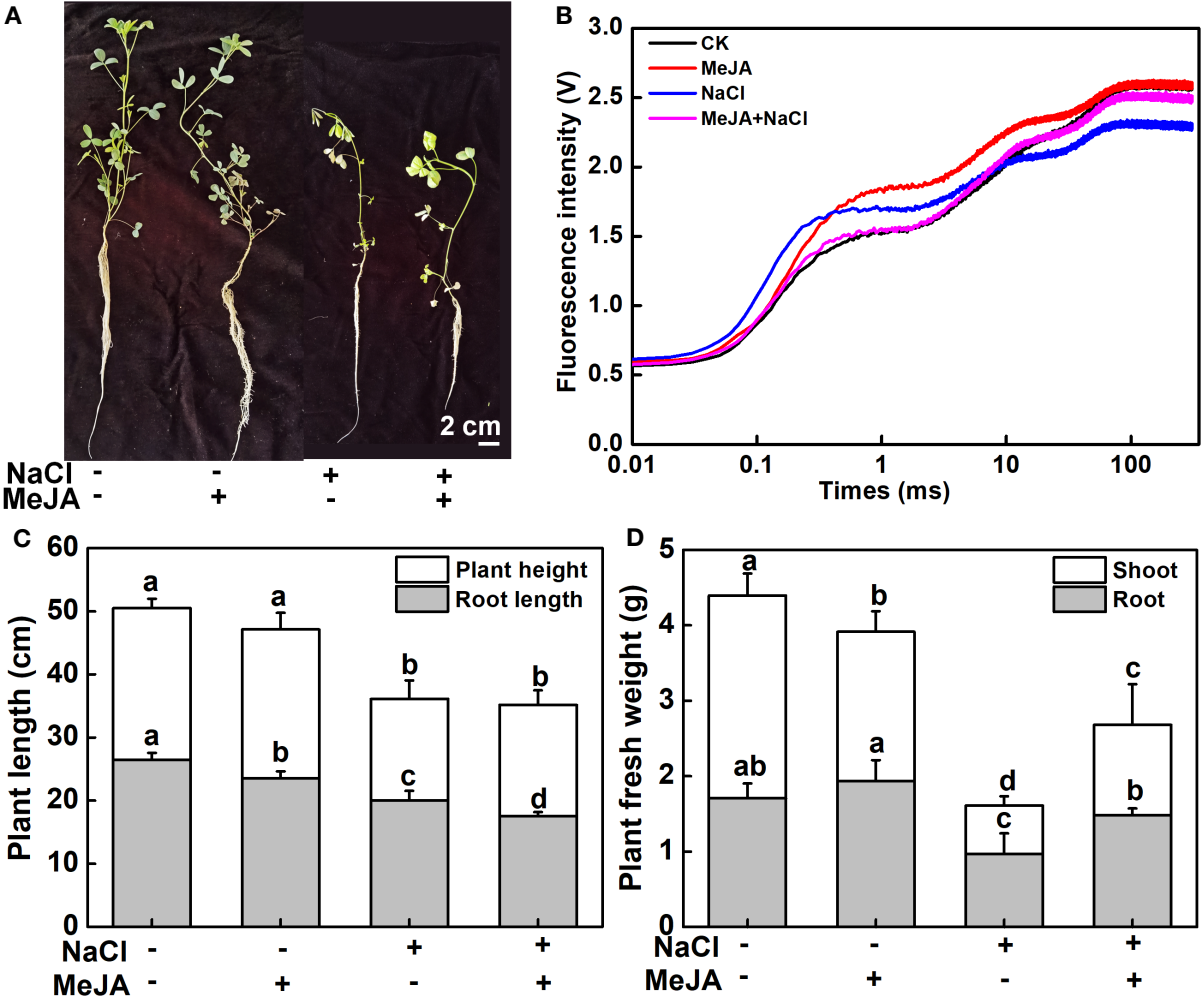
Exogenous methyl jasmonate (MeJA) enhances the salt tolerance of young alfalfa seedlings. **(A)** Phenotypes of young seedlings under NaCl treatment with or without exogenous MeJA. Bar = 2 cm. **(B)** Chlorophyll *a* fluorescence transient curves of leaves. CK indicates control. **(C)** Shoot and root length. **(D)** Shoot and root fresh weight. Columns represent average of at least six biological replicates and standard deviation. Columns with different lowercase letters indicate significant differences among treatments at *p* < 0.05 using Duncan’s test.

Further exploration was carried out to examine the influence of MeJA on the salt tolerance of young seedlings. As shown in **Figure 2A**, plants treated with a combination of MeJA and NaCl showed reduced leaf wilting compared to those treated with NaCl alone. Both NaCl treatment and NaCl combined with MeJA treatment decreased the OJIP fluorescence transient curve in leaves compared to the control group. However, the OJIP transient curve under the combined treatment was superior to that under NaCl treatment alone (**Figure 2B**). In addition, NaCl stress significantly inhibited plant growth, as indicated by the lower plant height and diminished root length in comparison to the control group. Interestingly, MeJA treatment significantly decreased the root length under normal conditions and further decreased when combined with NaCl treatment, compared to NaCl treatment alone (**Figure 2C**). This suggested that MeJA treatment can exacerbate the adverse impact of NaCl stress on root elongation. However, MeJA application alleviated the inhibition of NaCl stress on plant biomass, as evidenced by the higher fresh biomass of plant shoot and root under MeJA and NaCl combined treatment, as compared to NaCl treatment alone (**Figure 2D**)

### MeJA promoted redox equilibrium and ion homeostasis under salt stress

**Figure 3:**
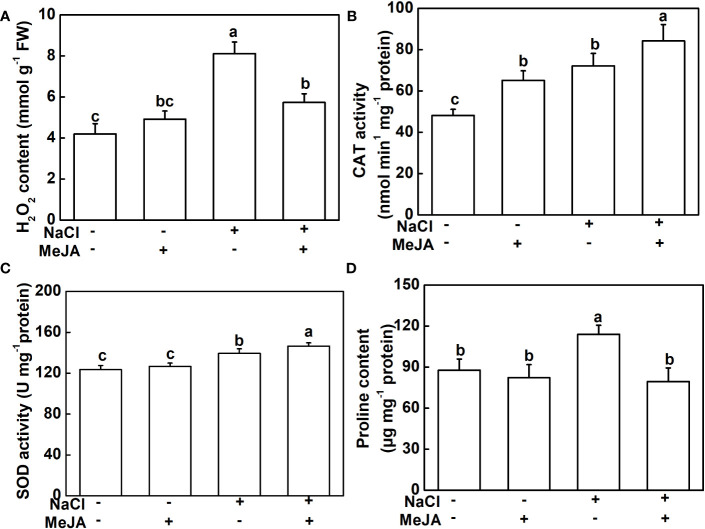
Exogenous methyl jasmonate (MeJA) alleviates the oxidative stress caused by NaCl treatment. **(A)** Content of H_2_O_2_. **(B)** Catalase (CAT) activity. **(C)** Superoxide dismutase (SOD) activity. **(D)** Content of proline. Columns represent average of three biological replicates with standard deviation, and each replicate has 10 plants. Different lowercase letters indicate significant differences among treatments at *p* < 0.05 using Duncan’s test.

To further explore the physiological mechanism underlying MeJA-enhanced salt tolerance, the antioxidant potential and ion content of roots under different treatments were measured. NaCl stress triggered a significant elevation in H_2_O_2_ and proline contents, along with heightened CAT and SOD activities in plant roots, compared to the control. However, plants treated with a combination of MeJA and NaCl exhibited a 25.6% decrease in H_2_O_2_ accumulation, when compared to those exposed to salt stress alone (**Figure 3A**). Furthermore, the combined treatment of MeJA and NaCl led to 16.7% and 5.1% increases in the activity of the antioxidant enzymes, CAT and SOD, respectively, compared to the NaCl stress group (**Figures 3B, C**). However, NaCl treatment promoted the production of proline, which could not be further induced by MeJA application, even causing a decrease in proline content compared to NaCl treatment alone (**Figure 3D**).

**Figure 4:**
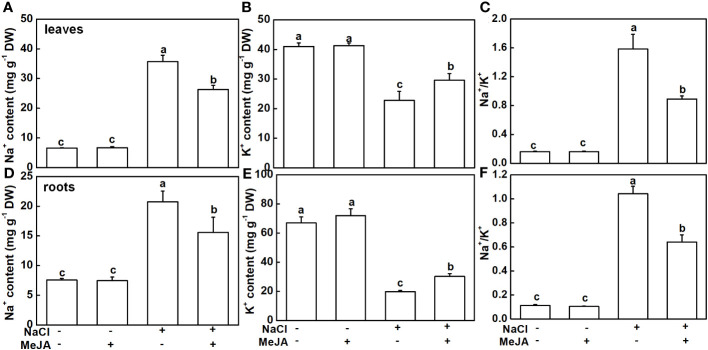
Effects of exogenous methyl jasmonate (MeJA) on Na^+^ and K^+^ contents under NaCl or non-NaCl treatment. **(A)** Content of Na^+^ and K^+^ **(B)** and Na^+^/K^+^ **(C)** in leaves. **(D)** Content of Na^+^ and K^+^ **(E)** and Na^+^/K^+^ **(F)** in roots. Columns represent average of three biological replicates with standard deviation, and each replicate has 10 plants. Different lowercase letters indicate significant differences among treatments at *p* < 0.05 using Duncan’s test.

The Na^+^ and K^+^ levels were detected in this study. As expected, NaCl stress caused a remarkable accumulation of Na^+^ and a decline of K^+^ in both leaves and roots, subsequently leading to higher Na^+^/K^+^ (**Figure 4**). MeJA alone did not result in distinguishable changes in Na^+^ and K^+^ levels, and Na^+^/K^+^ in leaves and roots (**Figure 4**). Importantly, MeJA addition effectively mitigated the negative effects of NaCl stress on Na^+^ and K^+^ homeostasis. Specifically, MeJA+NaCl treatment resulted in a notable reduction of Na^+^ accumulation by 26.2% in leaves and 25% in roots. Moreover, K^+^ levels exhibited a substantial increase of 30% in both leaves and roots, in stark contrast to the group subjected to salt stress without MeJA intervention (**Figure 4**). As a result, leaves and roots treated by MeJA+NaCl displayed lower levels of Na^+^/K^+^, when compared to those treated by NaCl alone (**Figures 4C, F**).

### Identification of genes regulated by MeJA in roots of alfalfa

**Figure 5:**
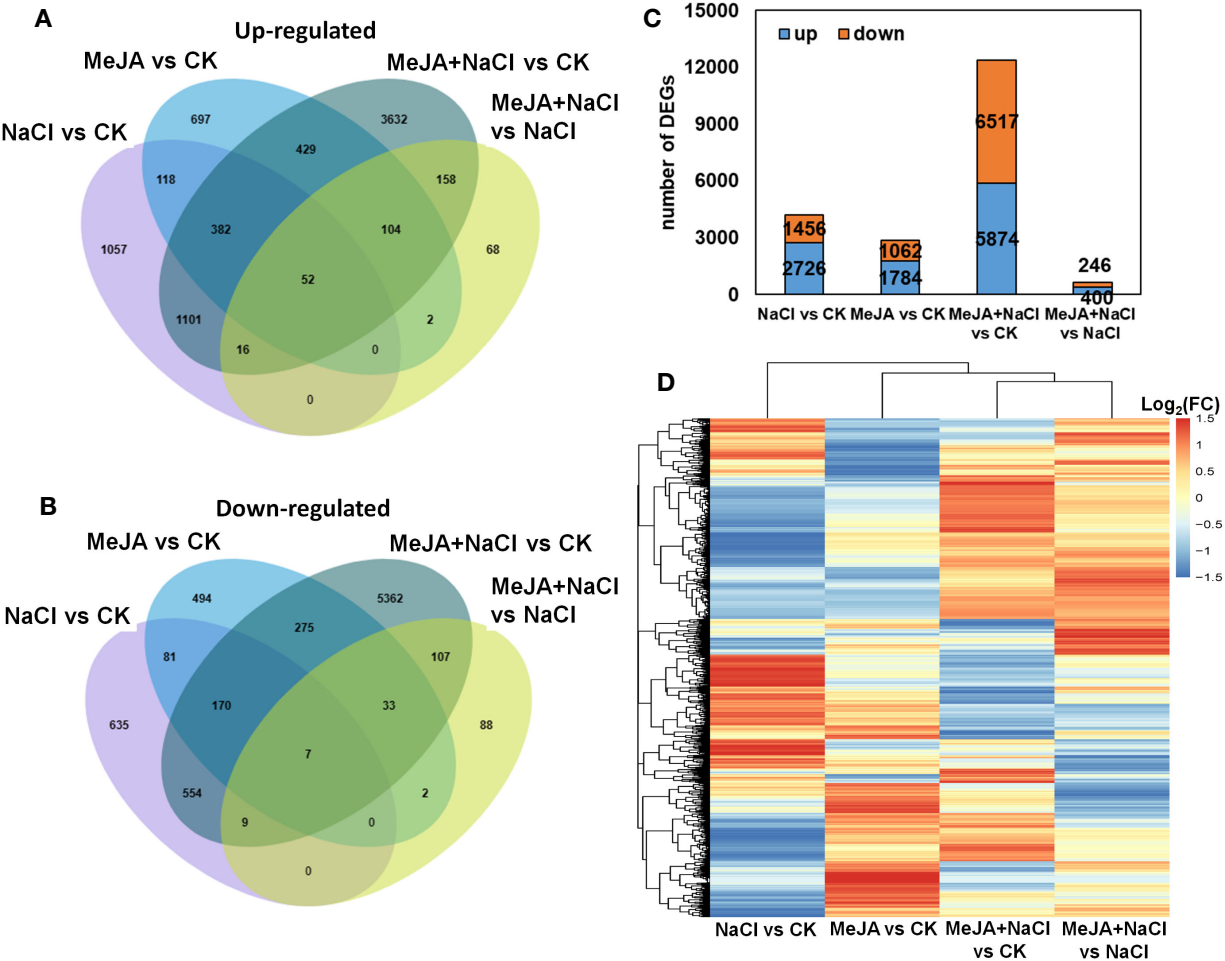
Integrated visualization of differentially expressed genes (DEGs) under control (CK), methyl jasmonate (MeJA), NaCl, and MeJA combined with NaCl (MeJA+NaCl). Venn diagrams show the overlap of upregulated **(A)** and downregulated genes **(B)**, indicating the unique and shared DEGs. **(C)** The count of DEGs in different comparisons. **(D)** A heatmap showing the log_2_ fold change (FC) of all DEGs across four treatment groups.

Furthermore, a genome-wide transcriptomic analysis of alfalfa roots under control (CK), MeJA, NaCl, and MeJA+NaCl was conducted. A total of 7,511 DEGs were identified by pairwise comparisons. Comparisons of NaCl vs. CK, MeJA vs. CK, MeJA+NaCl vs. CK, and MeJA+NaCl vs. NaCl consisted of 2,726, 1,784, 5,874, and 400 upregulated genes, respectively, and 1,456, 1,062, 6,517, and 246 downregulated genes, respectively (**Figures 5A–C**; **Table S2**). Importantly, there were 1,551 upregulated genes, and 740 downregulated genes were commonly regulated by both NaCl and MeJA+NaCl, compared to CK (**Figures 5A, B**). Among these shared genes, 68 exhibited upregulation while 15 displayed downregulation in in MeJA+NaCl vs. NaCl comparison, indicating that these genes were further induced or inhibited by MeJA based on NaCl treatment (**Figures 5A, B**). Moreover, the MeJA+NaCl vs. NaCl comparison revealed 332 upregulated genes and 230 downregulated genes, which were not detected in the NaCl vs. CK comparison, indicating that these genes were regulated by NaCl and MeJA+NaCl in an opposite direction (**Figures 5A, B**). A heatmap by clustering all DEGs was constructed to further depict the log_2_ (FC) in different comparisons and showed the different regulatory patterns by each treatment (**Figure 5D**).

### Functional categorization of the DEGs

**Figure 6:**
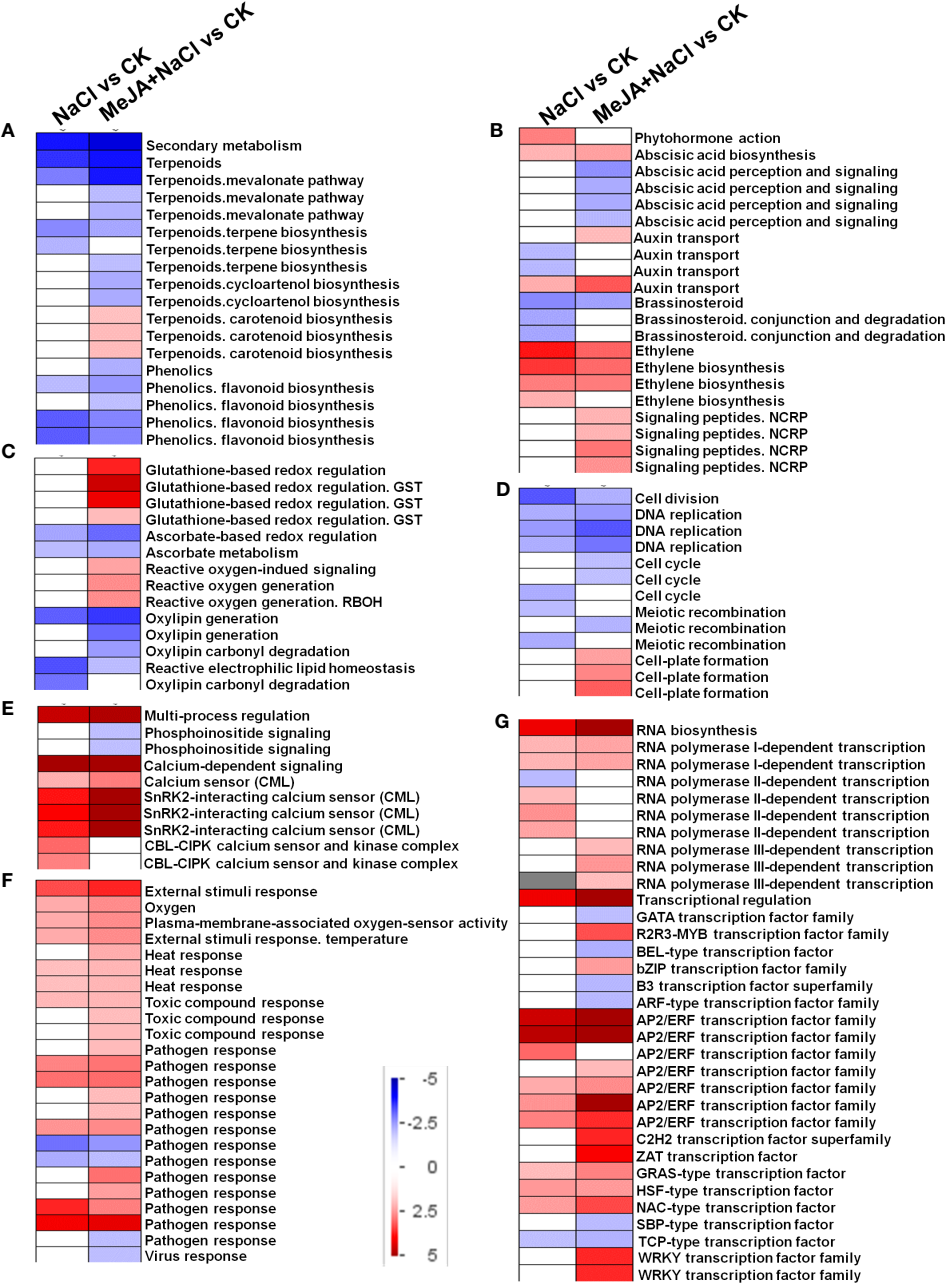
PageMan visualization of coordinated gene category changes modulated by NaCl and combined NaCl with methyl jasmonate (MeJA) (MeJA+NaCl). **(A)** Secondary metabolism. **(B)** Phytohormone action. **(C)** Glutathione-based redox regulation. **(D)** Cell division. **(E)** Multi-process regulation. **(F)** External stimulus response. **(G)** RNA biosynthesis. The log_2_ fold change (FC) in NaCl vs. CK and MeJA+NaCl vs. NaCl comparisons were subjected to over-representation analysis.

DEGs identified from NaCl vs. CK and MeJA+NaCl vs. CK comparisons were subjected to PageMan analysis to explore significantly over-represented functional pathways. As depicted in **Figure 6A**, the majority of sub-bins associated with secondary metabolism were down-enriched by both NaCl and MeJA+NaCl treatments, with the latter causing a more pronounced reduction in these sub-bins. Notably, the carotenoid biosynthesis category deviated from this trend, as it was significantly induced by the combined NaCl+MeJA treatment (**Figure 6A**; **Table S3**). Some gene categories enriched in phytohormone action were significantly upregulated in MeJA+NaCl vs. CK comparison, including genes involved in auxin transport (auxin efflux carrier component (*PIN*); auxin efflux transporter (*PILS*)) and signaling peptides (genes encoding *SCREW* precursor polypeptides and receptors) (**Figure 6B**; **Table S3**). Specifically, among the seven auxin transport genes, five exhibited higher expression levels under MeJA+NaCl treatment compared to their expression levels under NaCl treatment alone (**Supplementary Figure S1**). For redox homeostasis regulation, NaCl+MeJA treatment up-enriched gene categories related to glutathione-based redox regulation (*glutathione S-transferase* (*GST*)) and ROS generation (*NADPH-oxidase*, named *RBOH*) (**Figure 6C**, **Table S3**). For the roles of MeJA in the modulation of root elongation under NaCl stress, some cell cycle-associated genes, such as *Cyclins*, were depleted under the NaCl+MeJA condition (**Figure 6D**, **Table S2**). Moreover, a series of genes that participated in calcium perception (calcium sensor (*CML*) and *SnRK2-interacting calcium sensor*) were significantly upregulated by both NaCl alone and NaCl+MeJA combination treatment, whereas the latter resulted in a higher induction of these genes (**Figure 6E**, **Table S3**). In addition, NaCl+MeJA altered the expression of more external stimulus-responsive genes (such as genes related to toxic compounds and pathogen response) compared to NaCl alone (**Figure 6F**, **Table S3**). Furthermore, the regulation of sub-bins involved in RNA biosynthesis, especially TF families, varied between NaCl and MeJA+NaCl treatments. Specifically, certain TFs were exclusively induced by MeJA+NaCl treatment, such as R2R3-MYB, bZIP, ZAT, and WRKY TFs. However, NAC and most AP2/ERF TFs were induced under both NaCl and MeJA+NaCl treatments, with the latter resulting in greater induction of these genes (**Figure 6G**, **Table S3**).

### Co-expression network of genes related to redox and ion balance

**Figure 7:**
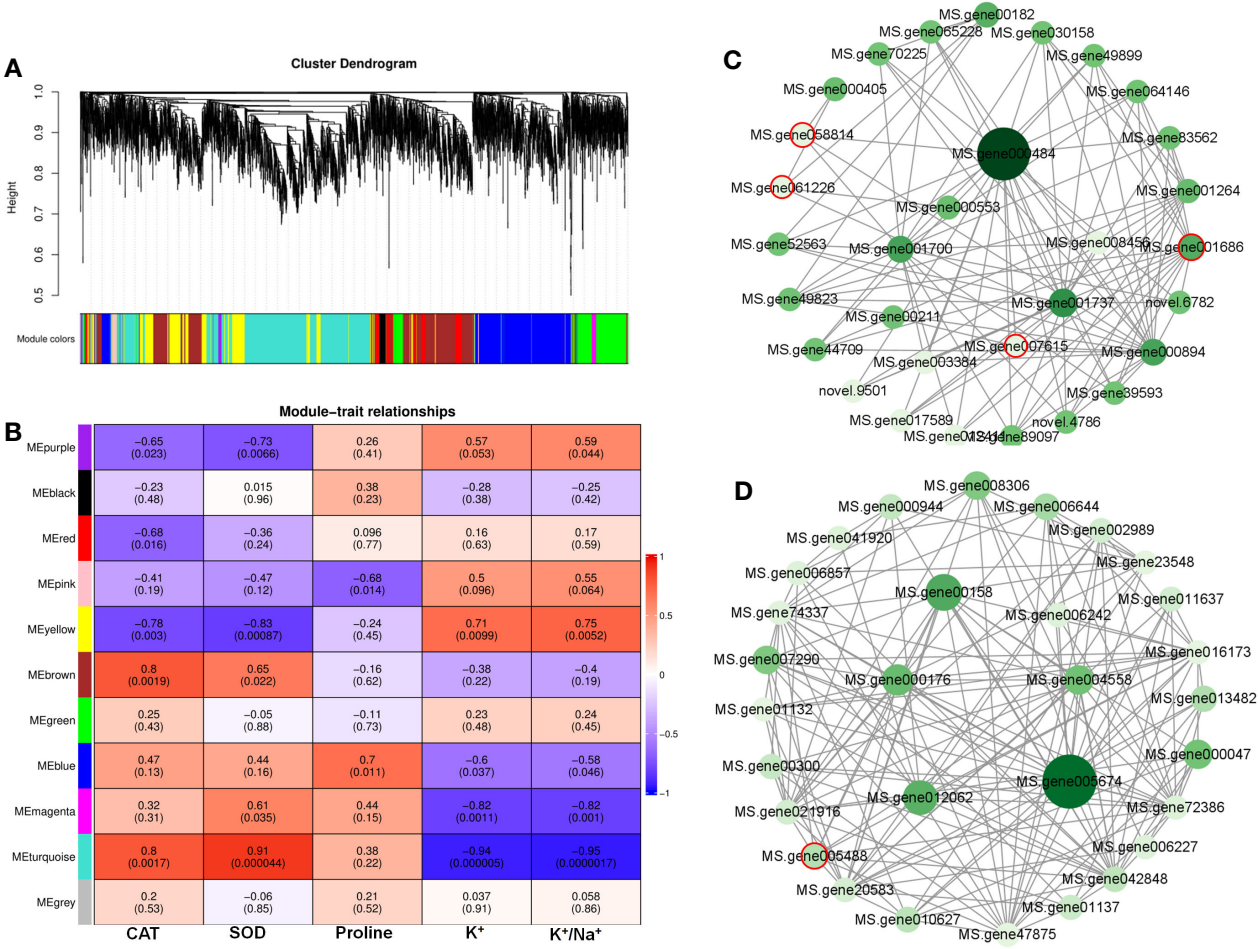
Weighted gene co-expression network analysis (WGCNA) reveals correlation between differentially expressed genes (DEGs) and salt stress-related physiological traits regulated by methyl jasmonate (MeJA). **(A)** Dendrogram of DEGs using WGCNA hierarchical clustering. **(B)** The thermogram shows the correlations between the modules and the physiological parameters. **(C)** The co-expression network of DEGs in MEturquoise. **(D)** The co-expression network of DEGs in MEyellow. Circles marked by red-colored edge lines indicate transcription factors.

A WGCNA was conducted to investigate the correlation between DEGs and physiological traits associated with salinity response. The analysis identified 11 co-expression modules (**Figure 7A**, **Table S4**). The module–trait analysis revealed a strong positive correlation between antioxidant capacity-related parameters (CAT and SOD activities) and transcription levels of genes in the turquoise module, with correlation coefficients ranging from 0.8 to 0.91 (**Figure 7B**). These results suggest that the genes in the turquoise module might have significant involvement in MeJA-enhanced antioxidant capacity under NaCl stress. Additionally, the eigengenes of the yellow module exhibited a significantly positive correlation with K^+^ content and K^+^/Na^+^, indicating their potential roles in MeJA-mediated ion transportation (**Figure 7B**).

**Figure 8:**
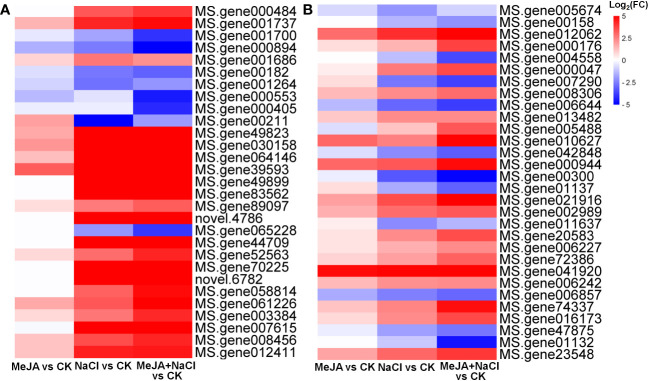
Heatmaps showing the log_2_ fold change (FC) of genes in turquoise **(A)** and yellow **(B)** modules.

Further, the turquoise module consisted of a total of 1,675 DEGs, whereas the yellow module contained 696 DEGs (**Table S4**). The top 30 genes according to the connectivity were characterized as hub genes in these two modules, which represent the integral function of the whole module (**Table S4**). Notably, most hub genes in the turquoise module were triggered by salt stress and showed the greatest expression level under MeJA+NaCl treatment (**Figure 8A**). The top 30 hub genes were visualized by Cytoscape software (**Figure 7C**). The top two hub genes encoding *pyruvate decarboxylase* (*PDC*; *MS.gene000484*) and *pyruvate kinase* (*MS.gene001737*) were identified to be involved in the Glycolysis/Gluconeogenesis process (ko00010) by Kyoto Encyclopedia of Genes and Genomes (KEGG) analysis (**Figure 7C**, **Table S5**). Moreover, this module contained four TF hub genes, including the GARP family encoding gene (MS.gene001686), AP2/ERF family encoding gene (MS.gene058814), MYB family encoding gene (MS.gene061226), and Trihelix family encoding gene (MS.gene007615). Among these TF hub genes, AP2/ERF and MYB were interconnected with *PDC*, and *GARP* was interconnected with *pyruvate kinase*. In addition, the most central hub gene in the yellow module was RNA demethylase *ALKBH10B* (*MS.gene005674*), homologous to *At4g02940* in *Arabidopsis* (**Figure 7D**; **Table S5**). The subsequent hub gene was ATP-citrate synthase beta chain protein, which was enriched in the tricarboxylic acid (TCA) cycle (ko00020) (**Figure 7D**; **Table S4**). A member of the MYB TF family (MS.gene005488) was listed as a hub gene in the yellow module (**Figure 7D**; **Table S5**). Most hub genes identified in the yellow module showed higher expression levels under MeJA+NACl treatment, compared to NaCl treatment alone (**Figure 8B**).

Finally, eight genes were randomly selected from the hub genes of turquoise and yellow modules for relative expressional validation by qRT-PCR (**Supplementary Figure S2**). The results were similar to the relative expression level (FPKM value) obtained from sequencing data, highlighting the reliability of our transcriptomic data.

## Discussion

### MeJA mediated root growth adaption of alfalfa under salt stress

MeJA exerted a suppressive effect on root elongation under both normal and salt stress conditions. This result aligns with previous studies involving JA-insensitive mutants, such as *jaz* and *coi*, which displayed a diminished response to root inhibition induced by JA (Chen et al., 2011; Geng et al., 2013). Moreover, salt stress-triggered JA signal suppressed cell elongation in the root elongation region of *Arabidopsis* (Valenzuela et al., 2016). *In vitro* experiments also evidenced that the G1 and G2 phases in the cell cycle of tobacco BY-2 cells were disrupted by JA application (Swiatek et al., 2004). Exogenous application of JA repressed root elongation, along with a reduction in root meristem size and expressional level of genes related to cell cycle (Chen et al., 2011). Consistently, MeJA+NaCl treatment caused a more significant downregulation of genes related to the cell cycle, implying that the inhibition of root elongation by JA under salt stress appeared to be influenced, at least partially, by the suppression of genes related to the cell cycle. Although MeJA inhibits root elongation, it leads to an increase in root biomass under salt stress, which might suggest the induction of lateral root formation by MeJA. This hypothesis was supported by that many JA-insensitive mutants exhibited impaired lateral root formation (Raya-González et al., 2012; Cai et al., 2014). JA was reported to directly induce the biosynthesis of auxin through its responsive transcription factor, ERF109, thus promoting lateral root development (Cai et al., 2014). Therefore, the upregulation of auxin transport genes regulated by MeJA under salt stress may contribute to the improvement of root architecture and facilitate better nutrient and water uptake.

Energy supply is decreased in salt-stressed plants because of the consumption of resources for osmotic regulation and inhibition of photosynthesis (Munns and Gilliham, 2015). Photosynthesis, both photosystem and carbon assimilation, is sensitive to salt stress (Kalaji et al., 2011). Chlorophyll *a* fluorescence transient is an effective criterion to estimate the impacts of abiotic stress on photosynthesis, and a decrease in OJIP was paralleled in the total carbon content in leaves (Li et al., 2017). Here, MeJA alleviated the inhibition of chlorophyll fluorescence transient curves caused by NaCl, which might be an important strategy to ensure energy supply. Taking into consideration the observed alterations in plant growth induced by MeJA, we propose that under salt stress conditions, MeJA prioritized the allocation of energy toward lateral root formation rather than overall plant elongation. This form modulation signified an adaptive strategy, wherein resources were strategically channeled to enhance resilience against salt-induced stressors. This hypothesize was supported by the research on *N. tangutorum*, which indicated that the amplification of JA signal by the addition of MeJA enhanced salt tolerance but aggravated salt-inhibited growth, along with the downregulation of growth-promoting genes (Gao et al., 2021).

### JA enhanced the salt tolerance of alfalfa by activating antioxidant enzymes and regulating ion transportation

Salt stress is widely acknowledged to induce oxidative stress, which causes severe damage to plants (Miller et al., 2010). JAs have been demonstrated to mitigate the negative impacts of oxidative burst by reducing ROS levels. Exogenous application of JA in wheat effectively promotes the antioxidant enzyme activity during salt stress (Zhu et al., 2022). Conversely, the JA-deficient mutant of tomato exhibited increased sensitivity to salt stress as a result of the depression of activity of antioxidant enzymes (Abouelsaad and Renault, 2018). Our study demonstrated that treatment with MeJA decreased H_2_O_2_ content and increased the activities of antioxidant enzymes SOD and CAT in roots exposed to salt conditions, together with upregulating specific genes encoding GST, an important enzyme in glutathione-based redox regulation. These findings provide further support for the involvement of JA in promoting plant antioxidant resistance under salinity conditions. In addition, a *PDC* gene was characterized as the hub gene in the module highly positively associated with antioxidant potential in our study. This gene showed the highest expression level under MeJA+NaCl treatment. A proteomic analysis revealed that pyruvate decarboxylase was induced by salt stimuli in rice (Damaris et al., 2016). ROS scavenging under salt stress requires enough energy supply, and alteration of genes related to sugar and starch metabolism may be critical to ensure the energy supply under abnormal conditions. Therefore, MeJA-enhanced antioxidant enzyme activity seems to be closely associated with *PDC*-mediated sugar metabolism.

High salinity results in the toxic accumulation of Na^+^ and impairs the uptake of K^+^, consequently perturbing ion balance (Yang and Guo, 2018). MeJA facilitated the maintenance of lower Na^+^ levels in both the root and shoot of salt-stressed plants by inducing Na^+^ efflux in *N. tangutorum* (Gao et al., 2021). As expected, MeJA was observed to decrease the Na^+^ content in NaCl-treated shoots and roots. However, K^+^ dissipations caused by NaCl in both roots and shoots were mitigated by MeJA application in our study. This finding contrasted with that obtained in salt-stressed *N. tangutorum*, which shows that MeJA improved K^+^ content only in shoots and reduced it in roots (Gao et al., 2021). Plants protect themselves from over-accumulation of Na^+^ mainly by inhibiting Na^+^ absorption by the root, excluding Na^+^ from the root, or reducing Na^+^ uploading in xylem sap (Zhu et al., 2016). Our results suggested that MeJA-mediated ion balance under salt stress might be dependent on the first two strategies, consequently relieving the inhibition in K^+^ uptake. In addition, we identified RNA demethylase gene *ALKBH10B* as one of the hub genes in the module highly related to ion balance. It is well known that the dynamic co-regulation of methylation and demethylation is a major process in regulating RNA transcription, and the demethylation was controlled by demethylase (Shi et al., 2022). *ALKBH* encodes a m6A demethylase, and some members belonging to *ALKBH* demethylase family were documented to be triggered by salt stress, such as *ALKBH9A* in *Arabidopsis* (Růžička et al., 2017). *Populus* plants overexpressed *PagALKBH9B* and *PagALKBH10B* showed enhanced salt resistance compared to the wild type (Zhao et al., 2022). In consequence, MeJA-regulated salt response in alfalfa may be involved in the participation of post-transcriptional regulation. The functions of these hub genes associated with antioxidant ability and ion balance need in-depth research in our further study.

### JA triggers other salt-responsive signals

Many second messengers, such as ROS and Ca^2+^, rapidly respond to salt stress and amplify salt response (Choudhury et al., 2013). The NADPH oxidase/respiratory burst oxidase homolog (*RBOH*s) play vital roles in controlling ROS production and participate in many defense-related signaling transduction (Liu et al., 2020). Transcript abundance of *RBOH* genes was proved to be upregulated by salt stress (Raziq et al., 2022). Grafting cucumber with pumpkin stock enhanced the Na^+^ exclusion under salt stress, which was attributed to the induction of NADPH oxidase (Niu et al., 2018). Additionally, changes in cytosolic calcium flux represent early signaling events under salt stress and guarantee the triggering of downstream signal transduction (Kaleem et al., 2018). An increase in calcium level is sensed by calcium-binding proteins, named calcium sensors, such as CML (Wang et al., 2018). The transcription of *CML*s was elevated by salt stimuli in many species (Sun et al., 2020; Li et al., 2022). Overexpression of an alfalfa *CML*, *MsCML46*, in tobacco enhanced plant salt tolerance by abating oxidative and osmotic stress (Du et al., 2021). These investigations strongly support the correlation between the initiation of ROS and Ca^2+^ and the activation of antioxidant systems and ion transport. Here, genes related to reactive oxygen-induced signaling and *RBOH* were specifically induced by MeJA+NaCl treatment. Also, many CML genes were upregulated by NaCl stress and further induced by MeJA+NaCl. These results suggest that MeJA application enhanced ROS and calcium signals under salt stress, which might contribute to the amplification of downstream salt-responsive signals and prime plants to defend against external salinity stimuli.

Numerous TFs are activated by salt stress to relay stress signals and govern the transcription of salt-responsive target genes by interacting with the *cis*-regulatory elements in promoters (Baillo et al., 2019). Recently, the identification and characterization of stress-responsive TFs have garnered significant interest. Some AP2/ERF TFs are JA-responsive, such as ERF109 in *Arabidopsis* (Cai et al., 2014) and ERF115 in *Salvia miltiorrhiza* (Sun et al., 2019). AP2/ERF TF were identified to act as favorable enhancers of plant salt tolerance, such as in tomato (Li et al., 2018), wheat (Rong et al., 2014), and *Arabidopsis* (Park et al., 2011). NAC TFs are plant-specific and participate in abiotic stress response, and transgenic soybean with overexpressed GmNAC06 exhibited heightened resistance to salt stress when contrasted with the wild type, attributable to their improved antioxidant ability and decreased Na^+^/K^+^ (Li et al., 2021). In this study, sub-bins associated with AP2/ERF and NAC TFs were upregulated by both NaCl and MeJA+NaCl treatment, with the latter resulting in a higher expression level of these TFs. Additionally, some TFs, such as the R2R3-MYB transcription factor family, WRKY, and ZAT, were not induced by NaCl alone but were remarkably increased by MeJA+NaCl treatment. Also, a MYB TF was identified as a hub gene positively related to ion balance. These results agreed with a transcriptomic analysis of wheat, which showed that R2R3-MYB and WRKY TFs were specifically induced only by MeJA+NaCl (Zhu et al., 2022). In *Arabidopsis*, overexpression of a *Malus domestica* C2H2-type zinc finger transcription factor, MdZAT17, enhanced plant salt resilience by reducing oxidative stress (Wang et al., 2022). Also, increasing evidence has documented that R2R3-MYB and WRKY TFs facilitated the conferral of salt resilience by manipulating the composite regulatory network of salt stress response (Ma et al., 2018; Du et al., 2022). Moreover, WRKY was reported to mediate JA-induced leaf senescence and was transcriptionally regulated by MYC2, the central TF in JA signaling (Wang et al., 2022). Therefore, the greater induction of these TFs responding to MeJA under salt stress could potentially contribute to the augmentation of salt tolerance in alfalfa.

In conclusion, our physiochemical and transcriptomic analyses revealed the roles of MeJA in mediating the salt tolerance of alfalfa. MeJA enhanced salt tolerance by rebuilding plant growth, improving antioxidant capacity, and maintaining ion homeostasis. MeJA addition to salt stress triggered a large-scale transcriptomic remodeling. Many genes related to signal transduction, such as second messengers and master TFs, were altered by MeJA under salt stress. The potential hub genes positively associated with salt-responsive traits are identified through WGCNA, suggesting that these genes might participate in MeJA-regulated salt tolerance. Taken together, we presented detailed evidence to deepen our insight into the molecular mechanisms underlying the role of JA in enhancing plant salt tolerance.

## Data availability statement

The original contributions presented in the study are publicly available. This data can be found here: https://www.ncbi.nlm.nih.gov/bioproject/PRJNA907206.

## Author contributions

YY: Data curation, Funding acquisition, Investigation, Validation, Writing – original draft, Writing – review & editing. TY: Investigation, Resources, Writing – original draft. SL: Investigation, Validation, Writing – review & editing. WW: Methodology, Software, Writing – review & editing. XL: Investigation, Writing – review & editing. SF: Conceptualization, Supervision, Writing – original draft, Writing – review & editing.
