# Supplementary figures and images for: Transcriptomic analysis reveals that methyl jasmonate confers salt tolerance in alfalfa by regulating antioxidant activity and ion homeostasis
Source: Front Plant Sci. 2023 Sep 14;14:1258498. doi: 10.3389/fpls.2023.1258498 (PMC10536279; doi:10.3389/fpls.2023.1258498)

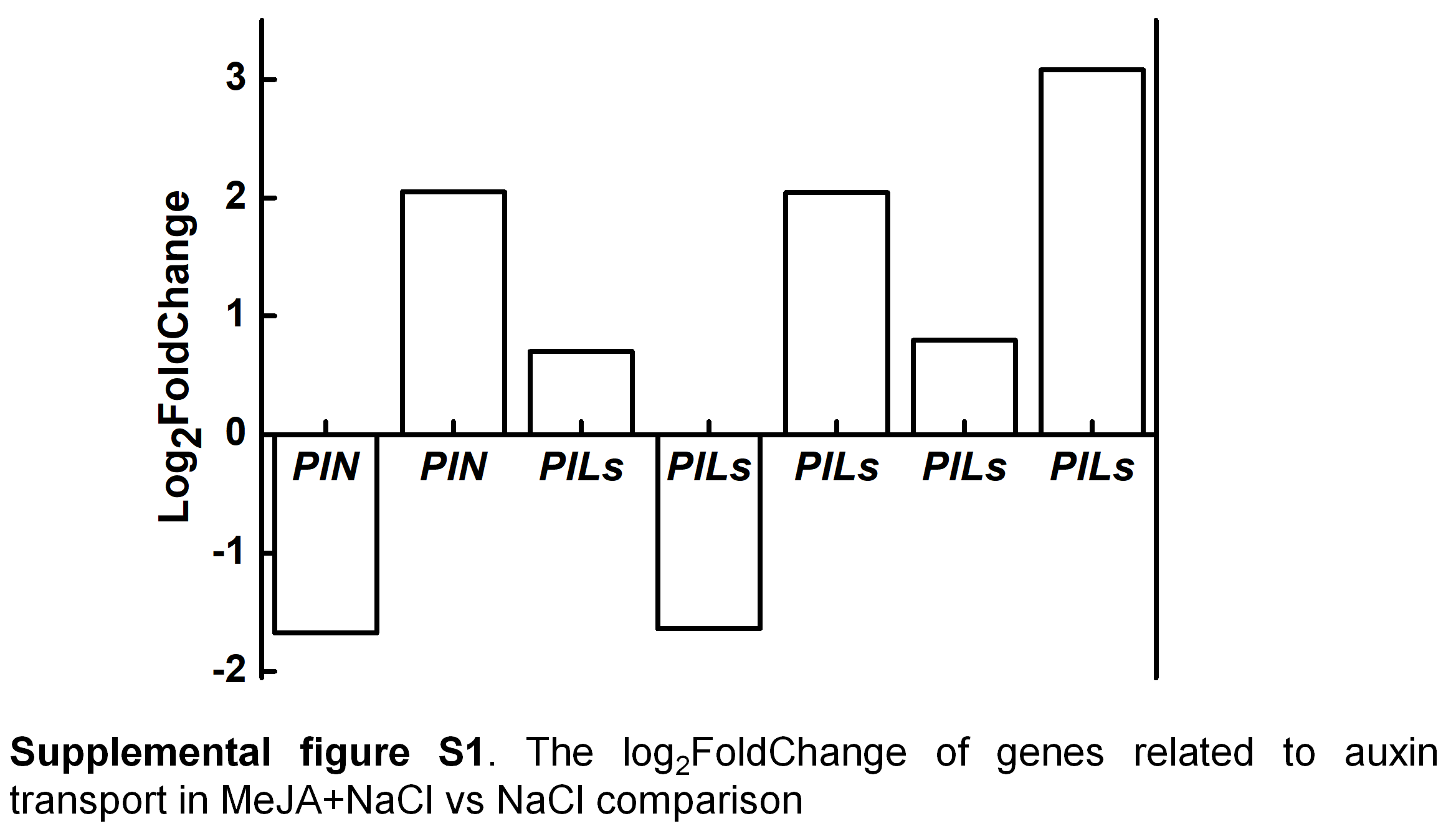

Supplement: Supplementary file 1 [file Image_1.tif]

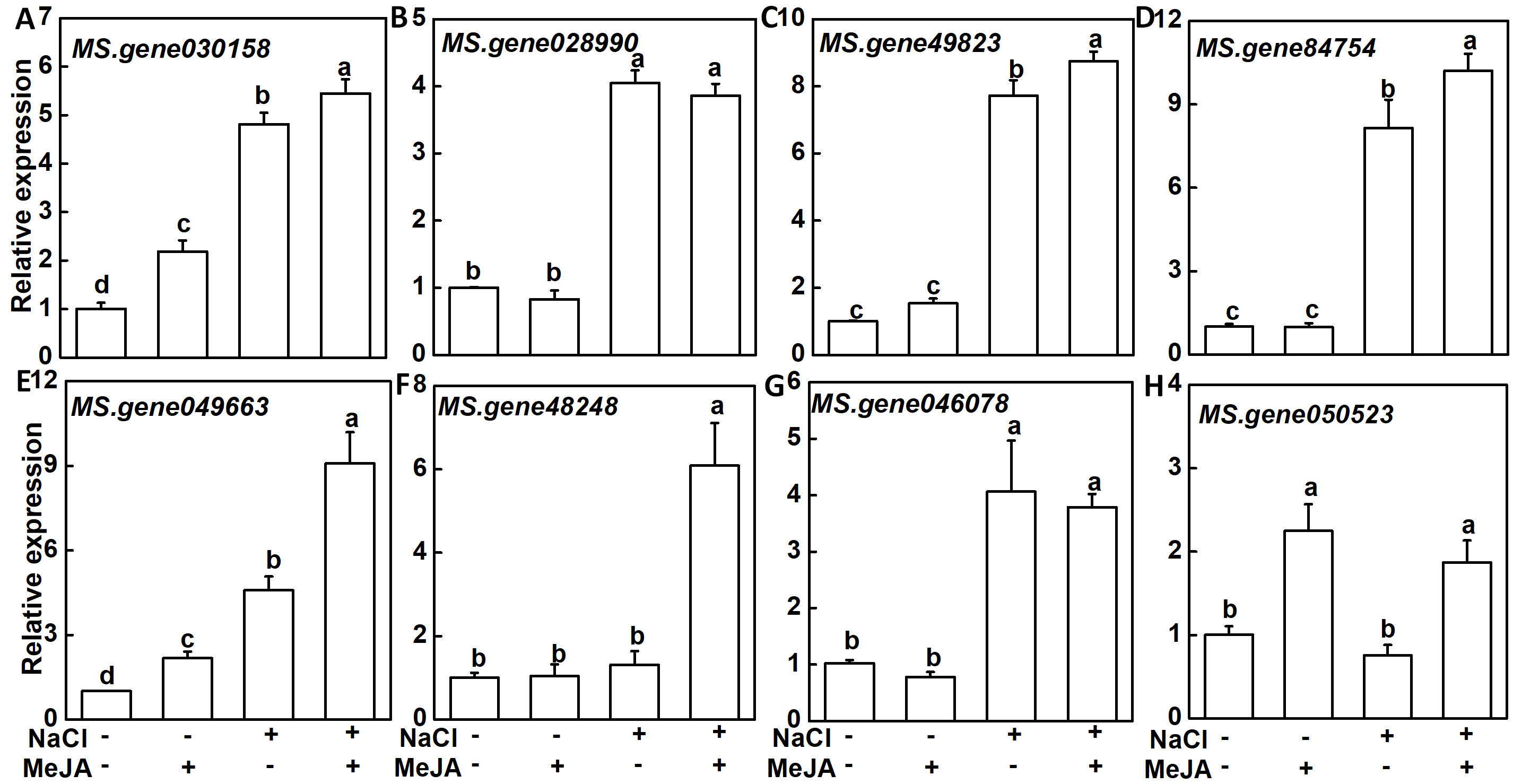

Supplement: Supplementary file 2 [file Image_2.tif]
