# Supplementary material for: Transcriptomic analysis reveals that methyl jasmonate confers salt tolerance in alfalfa by regulating antioxidant activity and ion homeostasis
Source: Front Plant Sci. 2023 Sep 14;14:1258498. doi: 10.3389/fpls.2023.1258498 (PMC10536279; doi:10.3389/fpls.2023.1258498)
Supplement: Supplementary file 3 [file Table_1.docx]

| Gene | Forward primers (5’-3’) | Reverse primers (5’-3’) |
| --- | --- | --- |
| *MS.gene000484* | AAAGCACCAAACAACGACGT | GAGGTTGAAGTCTCCGGGAA |
| *MS.gene030158* | GGAAAGAAGCCGCAGAAACA | GCAGTTTCCTTGGACGTCTG |
| *MS.gene058814* | AAGTCAAGTACCGAGGCGTT | AGCCAGAACCAGAACCAGAA |
| *MS.gene061226* | AGGAAGAGGAAGCGACAACA | TACCGGTTCGTTTGAGACCA |
| *MS.gene005674* | TGCTAACCCGTATGCTTTGC | AACTCCAGTACCACCACCAG |
| *MS.gene20583* | GCTGACCCTCAAACACGTTT | CAGGCGGCACAATCAGAATT |
| *MS.gene005488* | ATTCAGCCCGCACAACAATT | ACCACTCTTCCCCTGTTCTG |
| *MS.gene016173* | TGCTGGCTTTCAATCATGGT | GCGAAGAAGGTGCTGAAGAA |
| *Actin* | CCGACCTCGTCATACTGGTG | TCTTCAGGAGCAACACGCAA |

**Supplemental Table S1.** Primers designed for qRT-PCR analysis
